# Prediction of intraoperative red blood cell transfusion in valve replacement surgery: machine learning algorithm development based on non-anemic cohort

**DOI:** 10.3389/fcvm.2024.1344170

**Published:** 2024-02-29

**Authors:** Ren Zhou, Zhaolong Li, Jian Liu, Dewei Qian, Xiangdong Meng, Lichun Guan, Xinxin Sun, Haiqing Li, Min Yu

**Affiliations:** ^1^ State Key Laboratory of Medical Genomics, National Research Center for Translational Medicine at Shanghai, Shanghai Institute of Hematology, Ruijin Hospital, Shanghai Jiao Tong University School of Medicine, Shanghai, China; ^2^ Department of Cardiovascular Surgery, Ruijin Hospital, Shanghai Jiao Tong University School of Medicine, Shanghai, China; ^3^ Department of Cardiovascular Surgery, Shanghai General Hospital, Shanghai Jiao Tong University School of Medicine, Shanghai, China; ^4^ Department of Cardiovascular Surgery, Shanghai East Hospital, Tongji University School of Medicine, Shanghai, China

**Keywords:** intraoperative transfusion, machine learning algorithm, prediction, non-anemic, valve replacement

## Abstract

**Background:**

Our study aimed to develop machine learning algorithms capable of predicting red blood cell (RBC) transfusion during valve replacement surgery based on a preoperative dataset of the non-anemic cohort.

**Methods:**

A total of 423 patients who underwent valvular replacement surgery from January 2015 to December 2020 were enrolled. A comprehensive database that incorporated demographic characteristics, clinical conditions, and results of preoperative biochemistry tests was used for establishing the models. A range of machine learning algorithms were employed, including decision tree, random forest, extreme gradient boosting (XGBoost), categorical boosting (CatBoost), support vector classifier and logistic regression (LR). Subsequently, the area under the receiver operating characteristic curve (AUC), accuracy, recall, precision, and F1 score were used to determine the predictive capability of the algorithms. Furthermore, we utilized SHapley Additive exPlanation (SHAP) values to explain the optimal prediction model.

**Results:**

The enrolled patients were randomly divided into training set and testing set according to the 8:2 ratio. There were 16 important features identified by Sequential Backward Selection for model establishment. The top 5 most influential features in the RF importance matrix plot were hematocrit, hemoglobin, ALT, fibrinogen, and ferritin. The optimal prediction model was CatBoost algorithm, exhibiting the highest AUC (0.752, 95% CI: 0.662–0.780), which also got relatively high F1 score (0.695). The CatBoost algorithm also showed superior performance over the LR model with the AUC (0.666, 95% CI: 0.534–0.697). The SHAP summary plot and the SHAP dependence plot were used to visually illustrate the positive or negative effects of the selected features attributed to the CatBoost model.

**Conclusions:**

This study established a series of prediction models to enhance risk assessment of intraoperative RBC transfusion during valve replacement in no-anemic patients. The identified important predictors may provide effective preoperative interventions.

## 1 Introduction

Cardiac surgery patients are prone to suffer from blood transfusion therapy. Firstly, cardiovascular patients have a higher incidence of perioperative anemia (1). Secondly, cardiac surgery easily encounters ongoing blood loss and hemodilution intraoperatively. Thus, allogeneic transfusion is the standard treatment for perioperative anemia in patients undergoing cardiac surgery, and a wide variety of blood products have saved most patients’ lives. Historical cohort studies have consistently shown that allogeneic transfusion, particularly when administered intraoperatively, is related to increased postoperative complications, including infectious disease, renal failure, acute lung injury, and neurological adverse events (2–4). Our previous cohort study also revealed that intraoperative red blood cell (RBC) transfusion significantly increased the risk of postoperative hypoxemia in non-anemic adults undergoing isolated valve replacements (5). Therefore, it is widely accepted that intraoperative transfusion can act as a trigger for adverse events in cardiac surgery.

With the rapid advances in artificial intelligence, data-driven machine learning algorithms are being used to predict diverse clinical outcomes (6–8). Importantly, machine learning models have been widely used for the prediction of events in the cardiovascular field as well (9, 10). Machine learning algorithms provides new tools to overcome challenges for which traditional statistical methods are ill-suited, especially for making classification predictions using very specific sets of input features. The prediction of blood transfusion is a very suitable project for artificial intelligence, which has already been gaining sophistication with the help of various machine learning algorithms (11). Accurate prediction of intraoperative transfusion contributes to patient blood management and the prevention of the potential risks after transfusion. Patient blood management in cardiac surgery will facilitate a reduction in the misuse of allogeneic blood transfusion (12), illustrating the patients who are most likely to undergo transfusion should benefit most from disciplined blood management strategy. Therefore, it is necessary to build predictive models in cardiac surgery patients.

Herein, we attempted to establish machine learning-based algorithms in depth for predicting intraoperative RBC transfusion by incorporating preoperative data of the non-anemic adults undergoing valvular replacements and to evaluate its advantages compared with the traditional linear model, providing a new paradigm for predicting blood transfusion in cardiac surgery patients.

## 2 Methods

### 2.1 Study design and ethics statement

We enrolled the consecutive patients aged 18 to 80 years undergoing valvular replacements at Shanghai General Hospital, School of Medicine, Shanghai Jiao Tong University between January 2015 and December 2020. Exclusion criteria for this study included: (1) hemoglobin levels ≤12 g/dl for females and ≤13 g/dl for males, (2) emergency surgery, (3) massive transfusion, (4) re-operation of valvular surgery, (5) combined coronary surgery, aortic surgery, or ablation surgery, and (6) left ventricular ejection fraction (LVEF) <35%.

This retrospective study was approved by the ethics committee of Shanghai General Hospital (Approval number: 2020KY223). All the research procedures were carried out in accordance with relevant guidelines and regulations. As the data involved in this study were recruited from electronic medical records and reported without personal identifiers, the need for informed consent from the patients was waived.

### 2.2 Transfusion criteria

Intraoperative RBC transfusion was primarily determined by a hemoglobin level below 8.0 g/dl or hematocrit less than 25% after cardiopulmonary bypass, as per the established criterion (13). In our study, we defined massive transfusion as the administration of 5 or more units of RBCs within 4 h (14, 15). This definition is based on evidence indicating that massive hemorrhage is often linked to significant trauma, abnormal anatomy, or surgical techniques (16). The transfusion of other blood components varied among surgeons based on the extent of blood loss and intraoperative coagulation and bleeding.

### 2.3 Data collection

Preoperative patient characteristics were extracted from medical records, encompassing demographic information (e.g., age, gender, BMI), past medical history, and findings from laboratory tests, electrocardiogram, and echocardiography. These details also included smoking/drinking habits, comorbidities such as hypertension, diabetes, cerebrovascular disease, and atrial fibrillation, New York Heart Association (NYHA) class, LVEF, and a variety of blood tests such as serum ferritin, albumin, and platelet count.

### 2.4 Construction of machine learning algorithms

The patients included in this study were categorized into two groups based on whether they received intraoperative RBC transfusion (IRT), referred as the IRT group and non-IRT group respectively. The entire dataset was then randomly split into a training set and a testing set using an 8:2 ratio. Basically, the training dataset was used for feature selection. Then, a random forest (RF) algorithm-based feature selection method, Sequential Backward Selection (SBS), was used to filter the variables. In detail, firstly the RF algorithm uses feature importance as a ranking criterion. Secondly, the SBS method removes the least important feature in a full feature subset until the desired number of features is obtained. Subsequently, we measured the performance of each RF model with varying numbers of variables by plotting their accuracy. Finally, we identified the optimal number of features based on the highest model accuracy.

Then, the proposed prediction models for binary classification include the advanced ensemble learning methods, consisting of both nonlinear and linear algorithms. Nonlinear models included decision tree classifier, RF classifier, extreme gradient boosting (XGBoost) classifier, categorical boosting (CatBoost) classifier, support vector classifier (SVC). In contrast, the linear model was logistic regression (LR). Basically, the unit of a RF is the decision tree. RF consists of hundreds or thousands of decision trees and integrates all the categorical voting results, designating the category with the most votes as the final output (17). Unlike the bagging idea of RF, the boosting algorithms improve the prediction power by converting several weak learners to strong learners, such as CatBoost and XGBoost, both of which are based on the Gradient Boosting Decision Tree (18). Different from the tree-based methods, SVC finds a hyper-plane that creates a boundary between the types of data, which is a supervised machine learning model that has higher speed and better performance with a limited number of samples (19).

After developing the models, we aimed to evaluate the predictive validity of various machine learning models. To achieve this, we calculated and compared the areas under the receiver operating characteristic curve (AUC) and the accuracy of the algorithms. Moreover, we analyzed precision, which is the fraction of true positive examples among those classified as positive by the model, and recall, which is the fraction of positive examples classified correctly by the model. We also used the F1-score, defined as the harmonic mean of the model's precision and recall, to determine the model's overall accuracy on the dataset. Finally, to ensure correct interpretation of the prediction model, we employed the SHapley Additive exPlanation (SHAP) values. These values provided consistent and locally accurate attribution for each feature, allowing us to determine not only the importance of each feature but also its positive or negative impact on predictions (20). As most machine learning models are often considered as “black boxes”, SHAP values can be particularly useful in interpreting their results.

### 2.5 Statistical analysis

Continuous variables were expressed as either mean and standard deviation or median and interquartile range depending on whether they followed a Gaussian distribution. Categorical variables were reported as frequency and percentage. For group comparison of continuous variables, the Student's *T*-test was used if they followed a Gaussian distribution, while the Mann–Whitney *U* test was used for non-Gaussian data. The difference in the distribution of categorical variables between groups was tested using either the chi-squared test or Fisher's exact test, as appropriate.

For statistical analyses, we used SPSS software (ver. 22.0, SPSS Inc., Chicago, IL, USA). Model development was carried out using Python (ver. 3.9.6) with the packages of *catboost (1.0.6), sklearn (3.0.0), shap (0.41.0),* and *xgboost (1.6.1).* Graphics were generated using Python (ver. 3.9.6) with the package of *matplotlib (3.5.2)*. A *P*-value <0.05 was considered statistically significant for a two-sided test.

## 3 Results

### 3.1 Characteristics of study population

**Figure 1:**
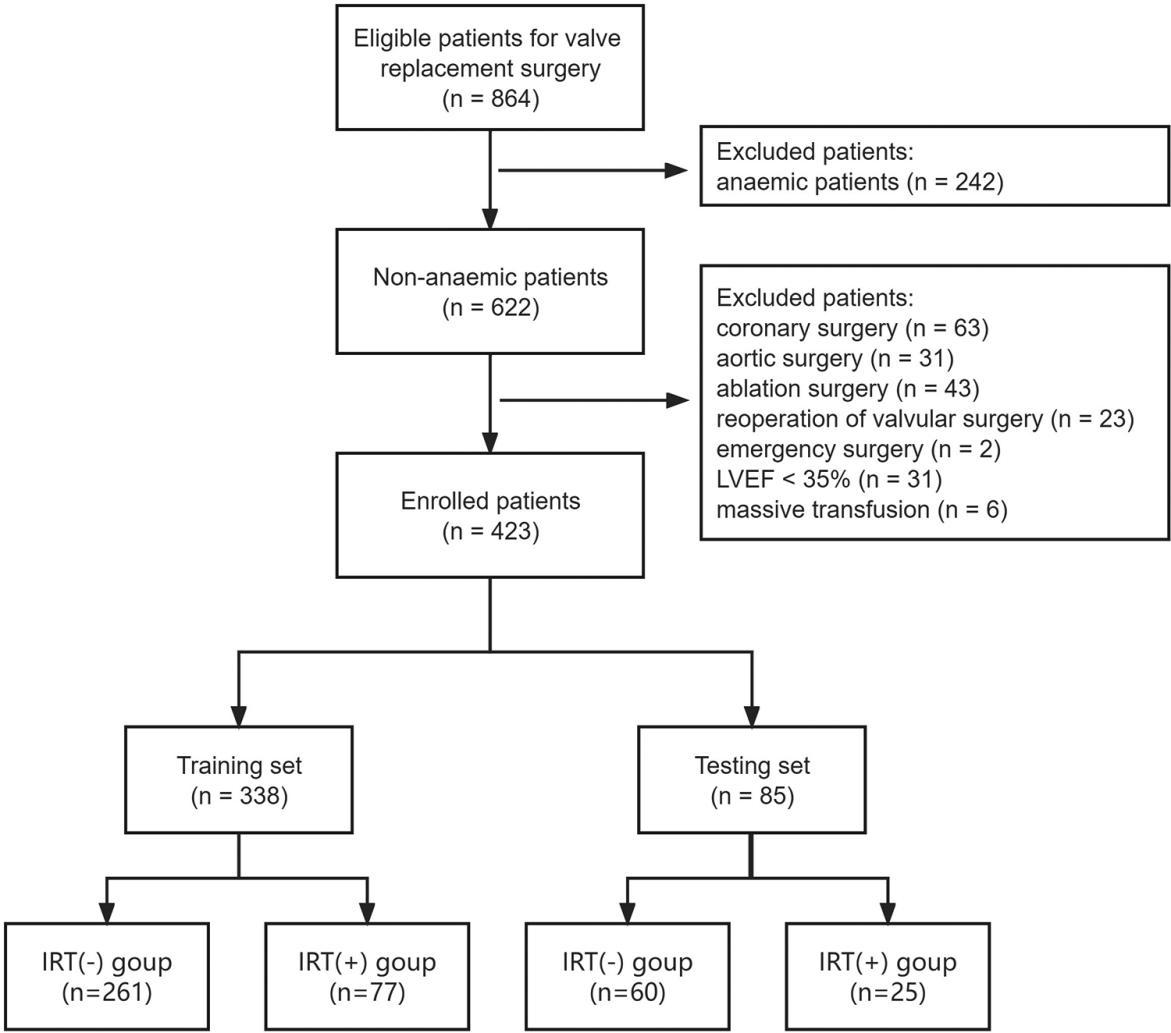
Workflow chart for recruitment in this study. LVEF, left ventricular ejection fraction; IRT, intraoperative red blood cell transfusion.

We finally enrolled the medical records of 423 consecutive patients undergoing isolated valvular replacements from January 2015 to December 2020. The structured workflow of the patient recruitment and grouping was presented in Figure 1. The patients had a mean age of 60.1 ± 11.4 years, and 102 participants (24.1%) received intraoperative RBC transfusion. They were randomly assigned to a training set (*n* = 338) or a testing set (*n* = 85). An overview over the preoperative patient data is given in Table 1. Each individual patient had 30 preoperative clinical characteristics. The incidences of intraoperative RBC transfusion were 22.8% and 29.4% in the training set and testing set, respectively.

**Table 1:** Patient characteristics and preoperative variables.

Clinical feature	Training set (*n* = 338)			Testing set (*n* = 85)		
	Non-IRT (*n* = 261)	IRT (*n* = 77)	*P*-value	Non-IRT (*n* = 60)	IRT (*n* = 25)	*P*-value
Male	168 (64.4)	23 (29.9)	<0.001	29 (48.3)	4 (16.0)	0.011
BMI (kg/m^2^)			0.960			0.809
<18.5	10 (3.8)	3 (3.9)		7 (11.7)	3 (12.0)	
18.5–25	137 (52.5)	38 (49.4)		27 (45.0)	9 (36.0)	
25–30	93 (35.6)	30 (39.0)		22 (36.7)	10 (40.0)	
≥30	21 (8.1)	6 (7.7)		4 (6.6)	3 (12.0)	
Age (years)			0.003			0.112
<60	125 (47.9)	24 (31.2)		30 (50.0)	11 (44.0)	
60–75	115 (44.1)	38 (49.4)		25 (41.7)	14 (56.0)	
≥75	21 (8.0)	15 (19.5)		5 (8.3)	0 (0.0)	
Hypertension	81 (31.0)	33 (42.9)	0.054	16 (26.7)	6 (24.0)	0.798
Diabetes	17 (6.5)	9 (11.7)	0.134	2 (3.3)	2 (8.0)	0.716
Smoking	20 (7.7)	3 (3.9)	0.370	8 (13.3)	1 (4.0)	0.375
Drinking	8 (3.1)	1 (1.3)	0.658	8 (13.3)	1 (4.0)	0.375
Stroke	15 (5.7)	5 (6.5)	0.807	5 (8.3)	3 (12.0)	0.905
Preoperative atrial fibrillation	78 (29.9)	32 (41.6)	0.055	16 (26.7)	12 (48.0)	0.057
Type of surgery			0.161			0.103
Isolated mitral valve replacement	127 (48.7)	39 (50.6)		30 (50.0)	16 (64.0)	
Isolated aortic valve replacement	63 (24.1)	21 (27.3)		14 (23.3)	5 (20.0)	
Isolated tricuspid valve replacement	27 (10.3)	3 (3.9)		3 (5.0)	2 (8.0)	
Mitral valve and aortic valve replacement	44 (16.9)	13 (16.9)		13 (21.7)	1 (4.0)	
Mitral valve, aortic valve, and tricuspid valve replacement	0 (0.0)	1 (1.3)		0 (0.0)	1 (4.0)	
PT (s)	13.71 ± 5.32	12.99 ± 2.90	0.125	13.92 ± 3.73	13.79 ± 4.06	0.888
APTT (s)	29.60 ± 5.72	30.37 ± 7.44	0.405	28.59 ± 4.87	28.21 ± 4.95	0.746
TT (s)	18.43 ± 3.60	18.30 ± 3.55	0.768	18.67 ± 3.38	19.38 ± 3.46	0.389
Fibrinogen (g/L)	2.70 ± 0.79	2.79 ± 1.03	0.491	2.71 ± 0.73	2.90 ± 1.02	0.355
ALT (U/L)	`32.22 ± 34.66	29.80 ± 29.11	0.577	30.57 ± 21.52	31.19 ± 26.87	0.911
Total bilirubin (µmol/L)	19.42 ± 12.69	17.03 ± 9.38	0.074	21.71 ± 25.45	17.40 ± 14.45	0.430
Serum creatinine (μmol/L)	80.20 ± 45.06	77.45 ± 22.81	0.606	81.13 ± 32.62	71.40 ± 15.36	0.065
eGFR [ml/(min·1.73 m^2^)]			0.063			0.159
≥120	38 (14.5)	3 (3.8)		11 (18.3)	1 (4.0)	
90–120	95 (36.4)	27 (35.1)		15 (25.0)	9 (36.0)	
60–90	102 (39.1)	38 (49.4)		22 (36.7)	12 (48.0)	
<60	26 (10.0)	9 (11.7)		12 (20.0)	3 (12.0)	
Hemoglobin (g/L)	138.79 ± 13.79	130.22 ± 12.94	<0.001	137.29 ± 14.12	129.77 ± 9.26	0.016
Blood type			0.843			0.977
A	84 (32.2)	27 (35.1)		19 (31.7)	9 (36.0)	
B	65 (24.9)	17 (22.0)		13 (21.7)	5 (20.0)	
O	91 (34.9)	25 (32.5)		22 (36.7)	9 (36.0)	
AB	21 (8.0)	8 (10.4)		6 (10.0)	2 (8.0)	
Platelet count (×10^9^/L)	170.92 ± 53.27	165.31 ± 66.58	0.499	181.38 ± 55.31	180.40 ± 57.58	0.941
Hematocrit (%)	41.74 ± 4.95	39.56 ± 4.11	<0.001	40.99 ± 4.42	39.96 ± 3.48	0.305
Vitamin B12 (pg/ml)	564.86 ± 309.34	628.45 ± 406.44	0.143	564.67 ± 386.72	514.68 ± 220.97	0.547
Folic acid (ng/ml)	8.44 ± 4.32	8.38 ± 4.57	0.904	8.81 ± 5.18	8.36 ± 3.91	0.697
Ferritin (ng/ml)	237.41 ± 187.47	184.87 ± 137.35	0.023	219.50 ± 142.08	170.41 ± 138.78	0.148
TAST (%)	31.50 ± 13.70	30.76 ± 13.75	0.679	29.12 ± 13.90	28.26 ± 12.46	0.791
LVEF (%)	57.70 ± 7.96	57.42 ± 9.71	0.812	56.92 ± 9.83	58.08 ± 7.42	0.597
Albumin (g/L)	37.18 ± 7.27	36.24 ± 9.71	0.320	36.90 ± 6.73	37.55 ± 6.00	0.488
HbA1c (%)	6.32 ± 1.29	6.44 ± 1.35	0.461	6.14 ± 0.94	6.39 ± 1.06	0.283
NYHA functional classification			0.693			0.326
≤2	35 (13.4)	9 (11.7)		5 (8.3)	0 (0.0)	
>2	226 (86.6)	68 (88.3)		55 (91.7)	25 (100.0)	

LVEF, left ventricular ejection fraction; BMI, body mass index; NYHA, New York Heart Association; PT, prothrombin time; APTT, activated partial thromboplastin time, TT, thrombin time; ALT, alanine transaminase; eGFR, estimated glomerular filtration rate; HbA1c, hemoglobin A1c; TAST, transferrin saturation; IRT, intraoperative RBC transfusion.

### 3.2 Machine learning predictive model development

**Figure 2:**
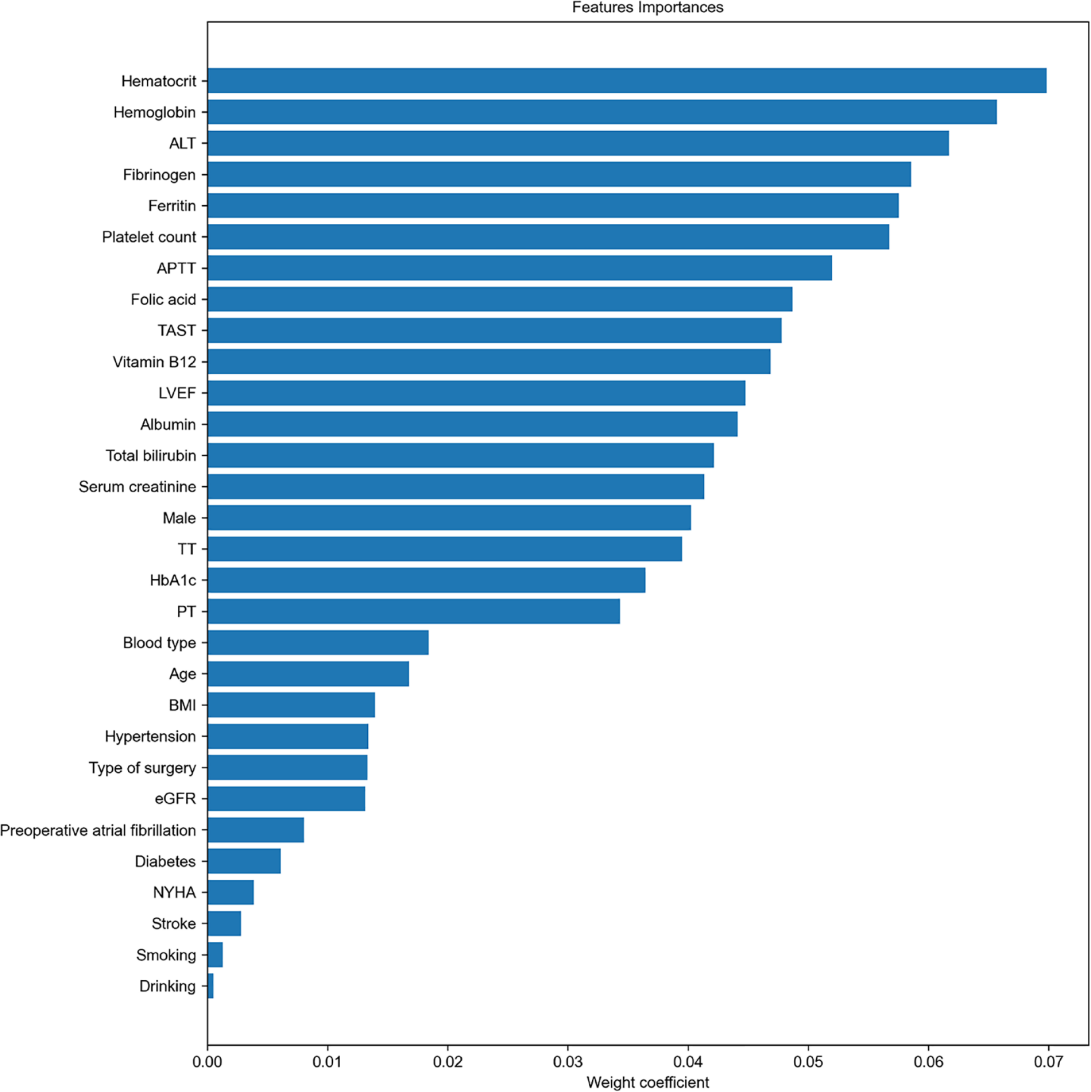
Importance matrix plot generated by the RF model to aid in feature selection. This plot provided a visual representation of the importance of each covariate and assisted in developing the final predictive model. LVEF, left ventricular ejection fraction; BMI, body mass index; NYHA, New York Heart Association; PT, prothrombin time; APTT, activated partial thromboplastin time, TT, thrombin time; ALT, alanine transaminase; eGFR, estimated glomerular filtration rate; HbA1c, hemoglobin A1c; TAST, transferrin saturation.

**Figure 3:**
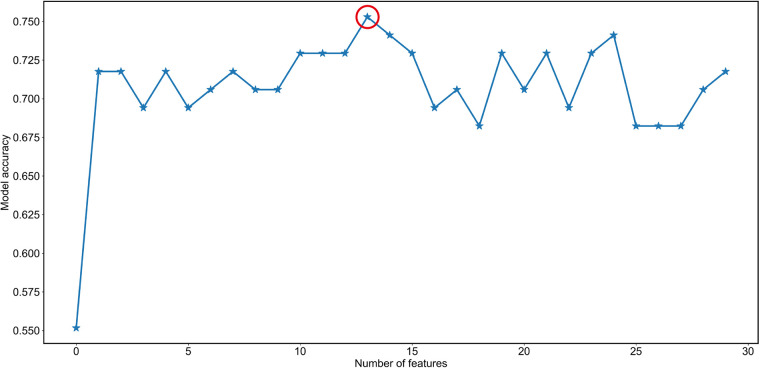
Feature selection by the Sequential Backward Selection (SBS) feature selection method. Firstly, the random forest algorithm was used to determine the importance of each feature. The features were then ranked in descending order based on their importance. Secondly, SBS was employed to remove the least important feature of the full feature subset until a new feature subspace with the desired number of features was obtained. Lastly, the optimal number of variables excluded from the model was determined by achieving maximum accuracy (indicated by the red circle). Specifically, 14 variables were deemed to be optimal for exclusion from the model.

A RF algorithm was applied to find the most critical variable associated with intraoperative RBC transfusion. The importance of each feature was ranked in descending order (Figure 2). The top 10 most significant preoperative feature listed in Supplementary Table S1 were hematocrit, hemoglobin, ALT, fibrinogen, ferritin, platelet count, APTT, folic acid, transferrin saturation (TAST), and vitamin B12. Afterward, all features were included in SBS one by one in order of their feature ranks. Based on the highest model accuracy (Figure 3), SBS excluded 14 features, illustrating that there were 16 important features incorporated in the subsequent machine learning models.

**Figure 4:**
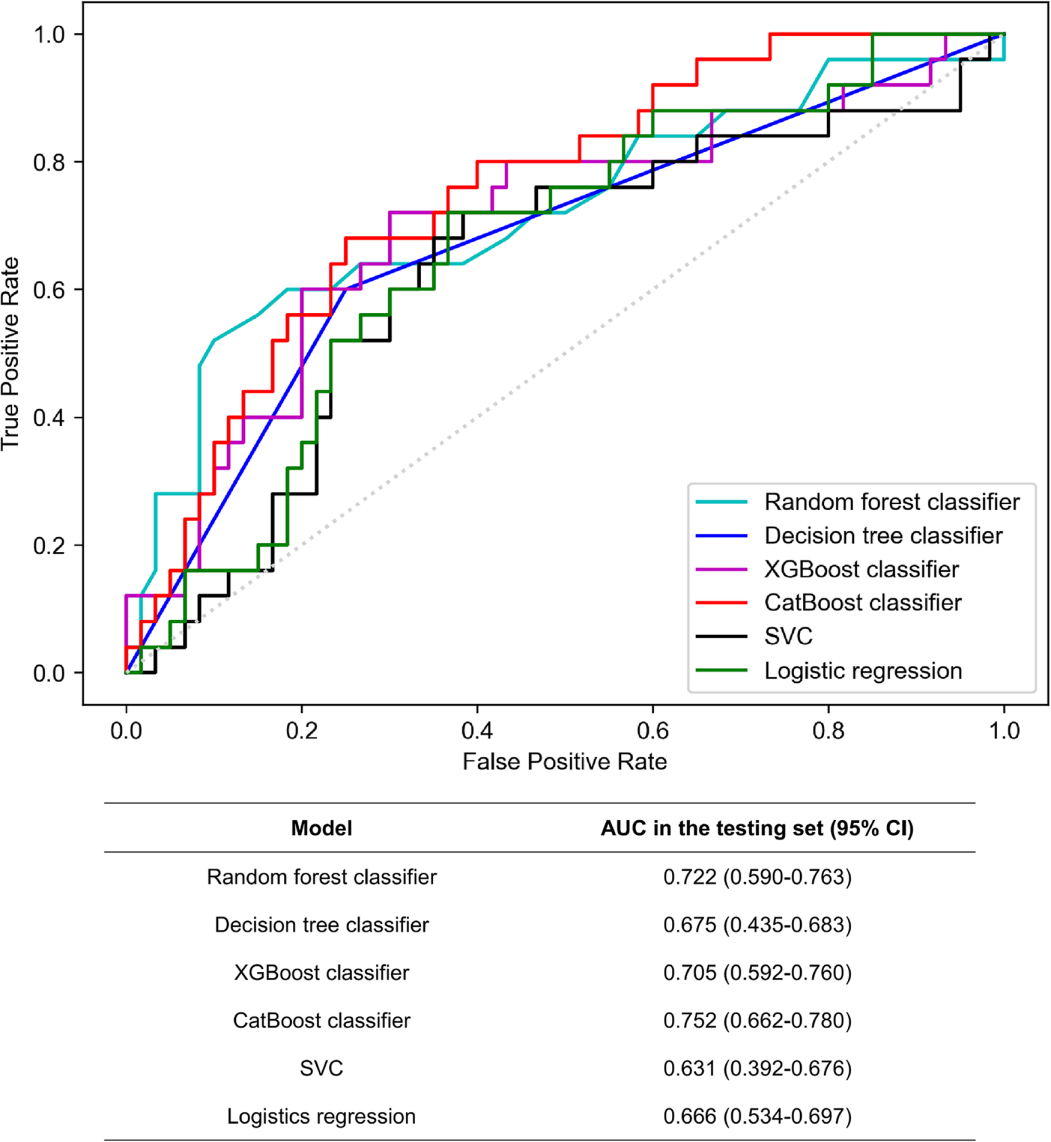
Comparison of receiver operating characteristic curves (AUCs) among machine learning models in the testing set. Among them, CatBoost displayed the most remarkable performance with the highest AUC value for predicting outcomes, demonstrating its potential in the field of predictive modeling. SVC: support vector classifier.

Using solely these 16 features, we trained six different models. As Figure 4 showed, the best AUC in the testing set were yielded by CatBoost method (AUC: 0.752), followed by RF model (AUC: 0.722) and XGBoost classifier (AUC: 0.705). In addition, RF classifier got the highest accuracy (accuracy: 0.734) and recall (recall: 0.728), while CatBoost method was slightly inferior (accuracy: 0.732 and recall: 0.725). CatBoost classifier also achieved decent F1 score (F1: 0.695), making the optimal model overall. Notably, the linear model, LR, was not effective enough and achieved the lowest recall, precision and F1 score among all the learning models. The detailed parameters of other algorithms are presented in Table 2.

**Table 2:** Performance of machine learning models.

Model	Accuracy	AUC	Recall	Precision	F1 score
XGBoost classifier	0.725	0.705	0.718	0.688	0.686
Random forest classifier	0.734	0.722	0.728	0.735	0.652
Decision tree classifier	0.606	0.675	0.675	0.725	0.713
CatBoost classifier	0.732	0.752	0.725	0.703	0.695
Support vector classifier	0.659	0.631	0.665	0.717	0.673
Logistics regression	0.635	0.666	0.625	0.684	0.650

AUC: receiver operating characteristic curve.

### 3.3 Machine learning-based model interpretation

**Figure 5:**
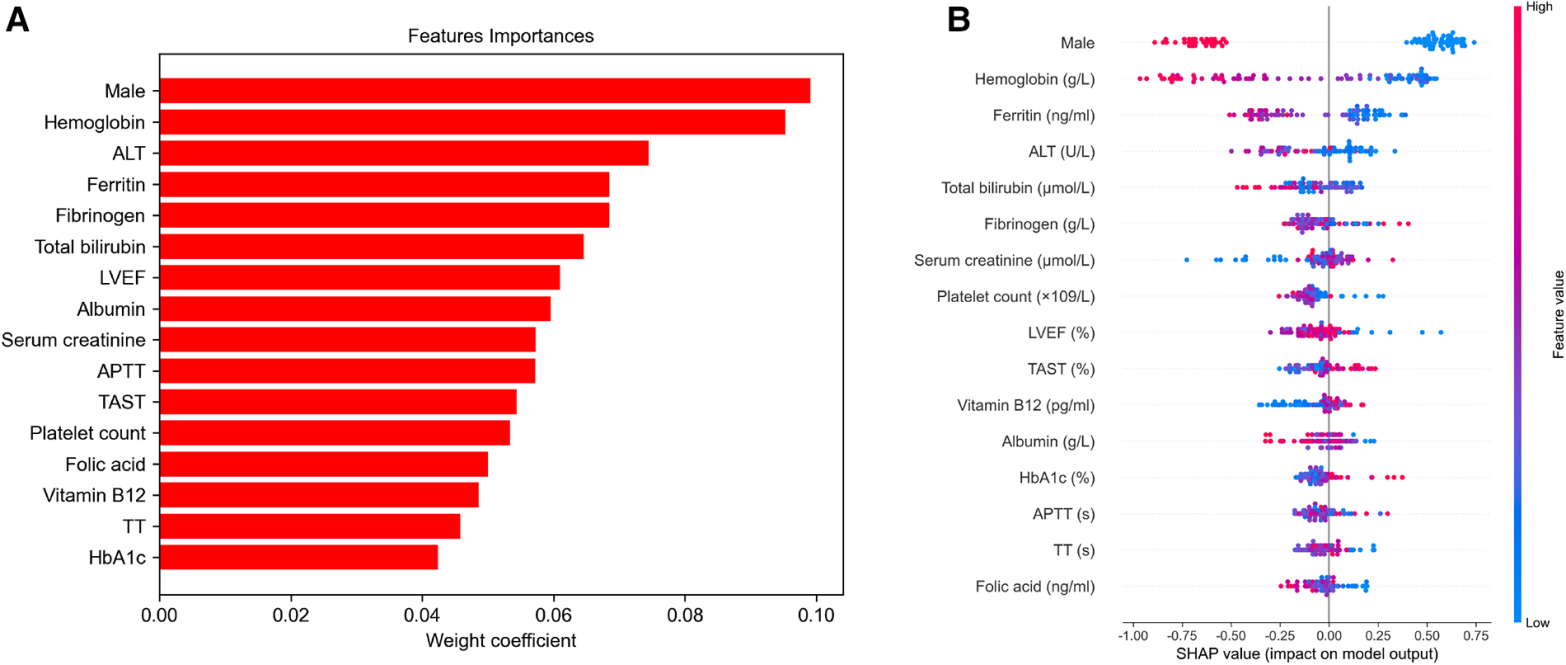
Feature importance and SHapley Additive exPlanation (SHAP) summary plot of the CatBoost model (**A**). The variables are ranked in decreasing order of their impact on the final model output (**B**). It is displayed that a dot estimation of the CatBoost model's output for each patient in the dataset. The color of each dot indicates the SHAP value of specific features; red denotes a higher SHAP value, while blue indicates a lower value. Higher SHAP values are associated with an increased risk of intraoperative red blood cell transfusion. LVEF, left ventricular ejection fraction; APTT, activated partial thromboplastin time; TT, thrombin time; ALT, alanine transaminase; TAST, transferrin saturation.

To identify the features that influenced the prediction model the most, the importance of selected feature in the CatBoost algorithm was calculated (Figure 5A). The importance of each feature was listed in Supplementary Table S2. Importantly, SHAP values were established to provide accurate attribution values for each feature within the CatBoost model. Finally, we depicted the SHAP summary plot of CatBoost algorithm (Figure 5B) and the SHAP dependence plot of all the features within CatBoost model (Supplementary Figure S1) to visualize the factors that facilitated intraoperative RBC transfusion.

## 4 Discussion

Given the strong association between intraoperative RBC transfusion and postoperative complications, predicting the need for such transfusions is critical to enabling early interventions that can address potential complications and improve patient prognosis following cardiac surgery. However, few studies have been conducted to predict intraoperative transfusion in cardiac surgery. To our knowledge, we were the earliest to establish the artificial intelligence algorithm for predicting intraoperative transfusion in non-anemic cohorts. As is known, nonlinear machine learning methods have remarkable superiority only if there is no way to fit the dataset linearly (21). Thus, we established five nonlinear machine learning models and one classical statistical regression model with preoperative characteristics. As we investigated, the optimal model, CatBoost algorithm, was superior to LR with an AUC of 0.752 (CatBoost) vs. 0.666 (LR), implying intraoperative RBC transfusion is not simply linearly related to preoperative risk factors based on our dataset. Undoubtedly, with the proficiency of artificial intelligence in handling nonlinear prediction tasks, more and more machine learning models are being used to predict transfusions in a series of different diseases, offering new approaches to improve clinical outcomes (22, 23).

Currently, machine learning technology is so advanced that it has given birth to many applications of predictive tasks in the field of medicine, which has the potential to revolutionize medical strategies and paradigms (24–26). However, there are still concerns about the overuse and misuse of machine learning in clinical research (27). The prejudice of machine learning algorithm mainly originates from the biased datasets, which is more common in the medical field. When the overall event rate is very low, machine learning models can easily build predictions with high accuracy and high AUC due to the high prediction rate for negative events. Mitterecker et al. (28) developed machine learning models of transfusion prediction based on large cohort using preoperative characteristics. When the overall event rate (transfusion) reached 12.4%, the models acquired not only very high accuracy, AUC, and negative predictive values, but also satisfactory recall and precision. On the contrary, accuracy and AUC of models still preserved a very high level but the recall and precision decreased sharply, when the overall event rate (massive transfusion) was only 0.4%. Interestingly, there are many measurements of model performance, but most of them do not show positive correlation, such as recall and precision. In fact, the performance of the model is closely related to the purpose of prediction. For the prediction of clinical outcomes, a higher prediction rate for positive events represents a lower rate of missed diagnoses, even if there are false predictions of negative events that can be further ruled out by other diagnostic methods. Historically, there have also been articles on predicting transfusion in cardiac surgery that sacrifice some performance metrics, such as recall and precision, to achieve high accuracy and AUC, which may not contribute much to the successful prediction of positive events from our point of view (29, 30). Therefore, in this study, we were not pursuing high accuracy and AUC of the models, but we try to build a more balanced model with improved precision and recall. For the biased datasets, we believe that the F1 score is more convincing for the evaluation of the model. Thus, based on the combination of AUC, recall, precision and F1 score, the CatBoost algorithm was selected as the optimal model even though the other methods were just slightly worse.

Of note, machine learning models are only a collection of predictive tools, as clinicians need to understand how they work. With SHAP values, the predictive pattern of the optimal machine learning model was fundamentally understood in our study and many risk factors, including female, low hemoglobin, low ferritin, low platelets, low folic acid, hypoalbuminemia, and high HbA1c, were detected. Actually, several factors have already been found to be associated with an increased risk of perioperative transfusion, such as the female gender and preoperative anemia (31). The anemic cardiac surgery patients have been reported to be more likely to get perioperative transfusion (31–33). It is of concern that low hemoglobin also played an important role in the prediction of positive events in non-anemic cohorts according to our results. However, there is no clinical evidence for direct intervention for relatively low hemoglobin levels in non-anemic patients. Interestingly, in previous work, we identified some factors that affect intraoperative RBC transfusion using LR model, such as female, hemoglobin, and the state of iron deficiency (5). Both linear and nonlinear models confirmed iron deficiency as a risk factor for intraoperative RBC transfusion, indicating that correcting preoperative iron deficiency might contribute to reducing the risk of intraoperative transfusion. Recently, a range of study found that preoperative intravenous iron treatment would significantly increase hemoglobin level (34) and reduce allogeneic transfusion (35) in non-anemic patients with iron deficiency undergoing cardiac surgery, supporting our findings about the impact of iron deficiency on non-anemic patients. In addition, another indicator that can be corrected preoperatively was hypoalbuminemia, which was reported as a risk factor of transfusion in other kinds of surgeries (36, 37). Of note, diabetes was not included in the machine learning model, while high HbA1c was adopted as a risk factor. Indeed, long-term high blood glucose levels can have detrimental effects on vascular health, potentially leading to increased vascular fragility in patients. This heightened vulnerability to intraoperative bleeding underscores the importance of closely monitoring and actively regulating perioperative blood glucose levels. By maintaining optimal blood glucose control during the perioperative period, healthcare providers can mitigate the risk of excessive bleeding and promote better patient outcomes.

Although our study provides valuable insights into predicting intraoperative RBC transfusion, it is important to acknowledge and consider certain limitations inherent in the research design. Firstly, the design was a single-center retrospective study with a small scale. The absence of an external validation set limited the accuracy of predictions. Consequently, before the models can be applied to external datasets, their predictive performance would require training and evaluation again. Secondly, our dataset is still a biased set. Continued inclusion of more patients and more characteristics is an effective way to increase the diversity of the datasets, addressing the bias. Thirdly, the certain data included in our study consists of continuous variables, which may not provide precise guidance for subsequent clinical applications. It is suggested that the establishment of additional criteria to convert these continuous variables into categorical variables, enabling their inclusion in the machine learning model for further analysis and refinement.

## 5 Conclusions

In summary, we have successfully identified and selected 16 crucial preoperative predictors for predicting intraoperative RBC transfusion following admission. Our study employed a range of cutting-edge machine learning algorithms, with the CatBoost classifier demonstrating the most robust performance for risk prediction. Furthermore, by leveraging the SHAP values from the CatBoost model, our team was able to identify key risk factors for intraoperative RBC transfusion in non-anemic patients, ultimately providing new insights and recommendations for improving cardiac surgical care.

## Data Availability

The raw data supporting the conclusions of this article will be made available by the authors, without undue reservation.
