# Supplementary material for: Prediction of intraoperative red blood cell transfusion in valve replacement surgery: machine learning algorithm development based on non-anemic cohort
Source: Front Cardiovasc Med. 2024 Feb 29;11:1344170. doi: 10.3389/fcvm.2024.1344170 (PMC10937389; doi:10.3389/fcvm.2024.1344170)
Supplement: Supplementary file 1 [file Table1.docx]

| **Features** | **Weight coefficient** |
| --- | --- |
| Hematocrit | 0.070 |
| Hemoglobin | 0.066 |
| ALT | 0.062 |
| Fibrinogen | 0.059 |
| Ferritin | 0.058 |
| Platelet count | 0.057 |
| APTT | 0.052 |
| Folic acid | 0.049 |
| TAST | 0.048 |
| Vitamin B12 | 0.047 |

Supplementary Table 1. Importance of the top 10 features in the prediction of the RF algorithm.

APTT, activated partial thromboplastin time; ALT, alanine transaminase; TAST, Transferrin saturation.

Supplementary Table 2. Importance of all the features in the prediction of the CatBoost algorithm.

| **Features** | **Weight coefficient** |
| --- | --- |
| Male | 0.099 |
| Hemoglobin | 0.095 |
| ALT | 0.074 |
| Ferritin | 0.068 |
| Fibrinogen | 0.068 |
| Total bilirubin | 0.065 |
| LVEF | 0.061 |
| Albumin | 0.059 |
| Serum creatinine | 0.057 |
| APTT | 0.057 |
| TAST | 0.054 |
| Platelet count | 0.053 |
| Folic acid | 0.050 |
| Vitamin B12 | 0.049 |
| TT | 0.046 |
| HbA1c | 0.042 |

APTT, activated partial thromboplastin time; TT, thrombin time; ALT, alanine transaminase; HbA1c, hemoglobin A1c; LVEF, left ventricular ejection fraction; TAST, Transferrin saturation.


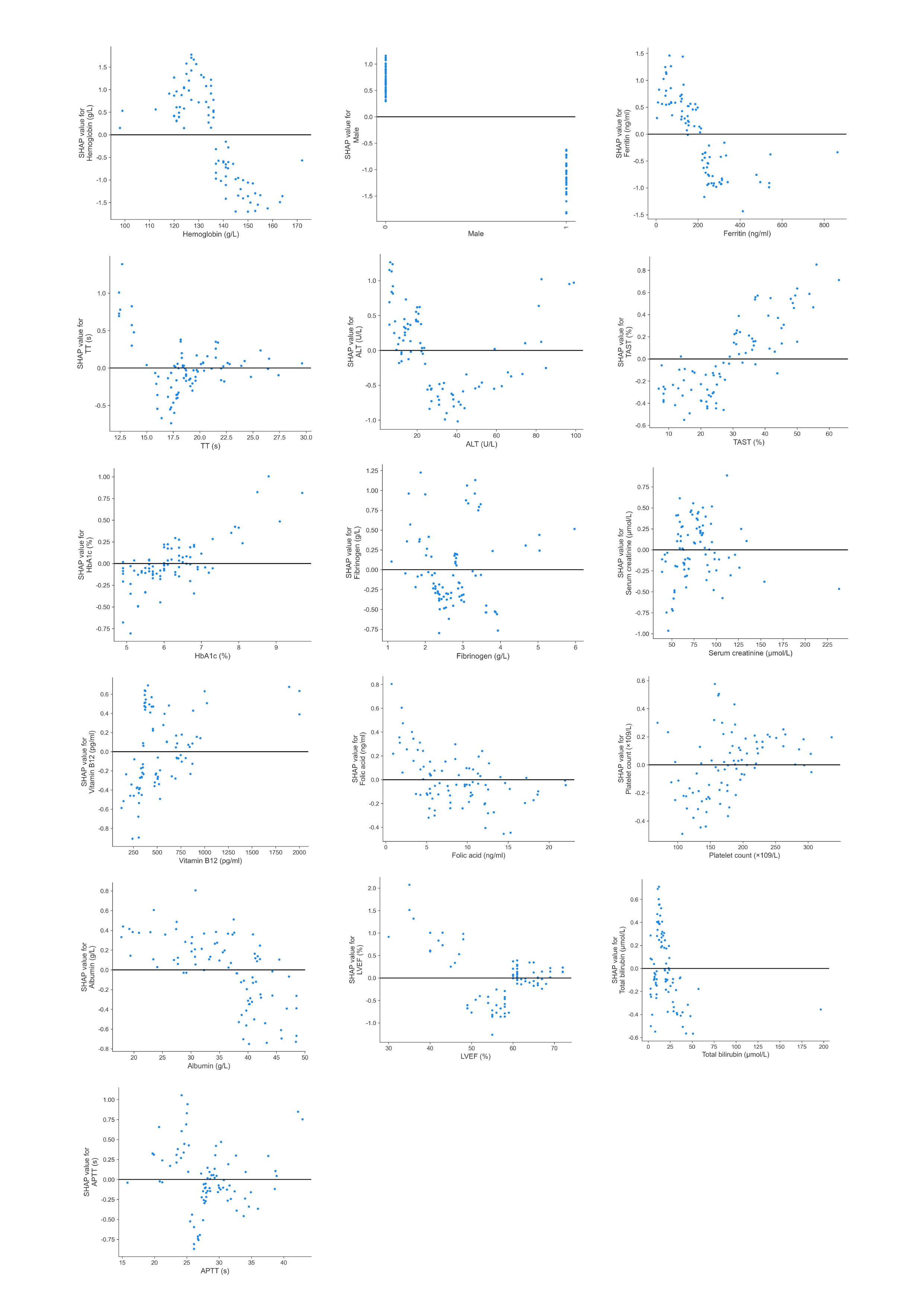


Suppementary Figure 1. SHAP dependence plot of the CatBoost model. In the SHAP dependence plot, each panel beautifully illustrates how every individual feature contributes to the output of the CatBoost prediction model. The x-axis elegantly displays the raw values of each feature while the y-axis gracefully indicates the SHAP values of the features. Moreover, when the SHAP value of a specific feature exceeds zero, it signifies an elevated risk of intraoperative RBC transfusion. Such a remarkable visualization enables easy interpretation and further analysis of the critical factors that impact the outcome of the prediction model. APTT, activated partial thromboplastin time; TT, thrombin time; ALT, alanine transaminase; HbA1c, hemoglobin A1c; LVEF, left ventricular ejection fraction; TAST, Transferrin saturation.
